# Hydrophilic Quantum Dots Functionalized with Gd(III)-DO3A Monoamide Chelates as Bright and Effective *T*_1_-weighted Bimodal Nanoprobes

**DOI:** 10.1038/s41598-019-38772-8

**Published:** 2019-02-20

**Authors:** Maria I. A. Pereira, Goreti Pereira, Camila A. P. Monteiro, Carlos F. G. C. Geraldes, Paulo E. Cabral Filho, Carlos L. Cesar, André A. de Thomaz, Beate S. Santos, Giovannia A. L. Pereira, Adriana Fontes

**Affiliations:** 1 Departamento de Biofísica e Radiobiologia, Universidade Federal de Pernambuco, Recife, PE Brazil; 2 Departamento de Química Fundamental, Universidade Federal de Pernambuco, Recife, PE Brazil; 3 Departamento de Ciências da Vida, Faculdade de Ciência e Tecnologia, Universidade de Coimbra, Coimbra, Portugal; 4 Centro de Química de Coimbra, Universidade de Coimbra, Coimbra, Portugal; 5 Departamento de Física, Universidade Federal do Ceará, Fortaleza, CE Brazil; 6 Departamento de Eletrônica Quântica, Instituto de Física Gleb Wataghin, Universidade Estadual de Campinas, Campinas, SP Brazil; 7 Departamento de Ciências Farmacêuticas, Universidade Federal de Pernambuco, Recife, PE Brazil

## Abstract

Magnetic resonance imaging (MRI) is a powerful non-invasive diagnostic tool that enables distinguishing healthy from pathological tissues, with high anatomical detail. Nevertheless, MRI is quite limited in the investigation of molecular/cellular biochemical events, which can be reached by fluorescence-based techniques. Thus, we developed bimodal nanosystems consisting in hydrophilic quantum dots (QDs) directly conjugated to Gd(III)-DO3A monoamide chelates, a Gd(III)-DOTA derivative, allowing for the combination of the advantages of both MRI and fluorescence-based tools. These nanoparticulate systems can also improve MRI contrast, by increasing the local concentration of paramagnetic chelates. Transmetallation assays, optical characterization, and relaxometric analyses, showed that the developed bimodal nanoprobes have great chemical stability, bright fluorescence, and high relaxivities. Moreover, fluorescence correlation spectroscopy (FCS) analysis allowed us to distinguish nanosystems containing different amounts of chelates/QD. Also, inductively coupled plasma optical emission spectrometry (ICP – OES) indicated a conjugation yield higher than 75%. Our nanosystems showed effective longitudinal relaxivities per QD and per paramagnetic ion, at least 5 times [per Gd(III)] and 100 times (per QD) higher than the *r*_1_ for Gd(III)-DOTA chelates, suitable for *T*_1_-weighted imaging. Additionally, the bimodal nanoparticles presented negligible cytotoxicity, and efficiently labeled HeLa cells as shown by fluorescence. Thus, the developed nanosystems show potential as strategic probes for fluorescence analyses and MRI, being useful for investigating a variety of biological processes.

## Introduction

Magnetic resonance imaging (MRI) and optical techniques have been applied as important tools to observe and investigate a variety of biological processes^1,2^. Optical methods, especially those based on fluorescence, are very sensitive, capable to detect even a single molecule with a spatial resolution down to 10 nm^3^. Unfortunately, the penetration depth of light beams does not allow the visualization of organs localized in deep regions of biological systems for *in vivo* studies^2^. MRI, on the other hand, is a non-invasive technique capable to trespass the whole body and discriminate healthy from pathological tissues, with a high anatomical resolution and definition. Nevertheless, the use of MRI contrast agents (CAs), which are able to decrease the nuclear relaxation times (*T*_1_ and *T*_2_) of surrounding proton water molecules, may also be required in order to obtain the desirable tissue differentiation. Paramagnetic chelates containing Gd(III) ions are commonly used clinically as *T*_1_-weighted MRI CAs^4^. However, even applying CAs, MRI still lacks both the sensitivity and specificity of fluorescence-based techniques. A bimodal fluorescent/paramagnetic nanoprobe, therefore, could be used to take advantages of both tools, and applied to study the fundamentals of diverse biological processes, such as those associated to diseases, in real-time and at different levels of biological organization^5,6^.

In this context, quantum dots (QDs) show a high potential for the development of successful bifunctional nanoprobes due to the unique properties of these semiconductor nanocrystals, such as: (i) a bright fluorescence and a great resistance to photodegradation, allowing to monitor long-term and real-time biological events and (ii) a highly active surface, enabling their conjugation not only with biomolecules, drugs, other nanoparticles, but also with paramagnetic compounds^7–11^.

Bimodal nanosystems based on QDs associated with superparamagnetic or paramagnetic compounds have been prepared by using different experimental strategies^12–21^. Among the paramagnetic compounds, the most explored involved the indirect attachment of Gd(III) chelates to QDs using lipid, polymeric or silica layers^12,13,15,17–19^. Nevertheless, bimodal nanoprobes, prepared by this strategy, can display some drawbacks. For instance, they: (i) cannot be so effective in reducing the *T*_1_ relaxation time, mainly due to the additional slow water exchange across the nanoparticle surface; (ii) cannot present a proper nanometer size for biological applications; and/or (iii) demand laborious preparation procedures^22,23^. Experimental approaches based on the direct conjugation of QDs and Gd(III) chelates have also been explored^24–26^, which promise to maintain both, a small system size, and an effective dynamic water exchange between the bound water molecules and bulk water, without barriers, allowing to reach more efficient *T*_1_ relaxation time reductions and, as consequence, higher *r*_1_ relaxivities. However, in general, these nanosystems are usually based on hydrophobic QDs and not always present both a bright fluorescence and improved relaxometric properties.

Therefore, herein we report the development of a new fluorescent/paramagnetic nanoparticulate bimodal system based on hydrophilic CdTe QDs directly conjugated by covalent bonds to Gd(III)-DO3A monoamide chelates. This Gd(III)-DOTA derivative chelate was chosen for the QDs conjugation due to the high thermodynamic and kinetic stability^27^ and good relaxometric parameters, such as a long electronic relaxation time (τ_S_)^28^, which make the original Gd(III)-DOTA chelate (Dotarem^®^), an important clinically applied MRI CA. For this, the stability of the chelate Gd(III)-DOTA-NHS (1,4,7,10-tetraazacyclododecane-1,4,7,10-tetraacetic acid mono-*N*-hydroxysuccinimide ester), obtained after activation by covalent coupling agents, was assessed using a transmetallation assay and the conjugation was evaluated by fluorescence correlation spectroscopy (FCS). Bimodal nanosystems were characterized by optical spectroscopy and relaxivity measurements, per ion and per QD at 20 MHz (25 and 37 °C) and 60 MHz (37 °C). Lastly, the cytotoxicity of the nanosystems was evaluated as well as their ability to label cervical adenocarcinoma (HeLa) cells by fluorescence. We believe that the designed nanoprobes are promising to be applied as tools for upcoming *in vivo* and *in vitro* studies based on fluorescence and MRI, aiming the comprehension of a variety of biological processes.

## Results and Discussion

### Coordination efficiency evaluation by the xylenol orange method

The evaluation of the absorbance ratio between the peaks at 577 and 434.5 nm of xylenol orange as a function of known gadolinium concentrations (Fig. S1A – Electronic Supporting Information - ESI**†**) originated a calibration curve (Fig. S1B - ESI**†**), which allowed us to quantify the amount of eventual free gadolinium ions remaining in the chelate sample^29^. According to the calibration curve (r^2^ = 0.99), we found an amount of 0.29 µM of free Gd(III) in the aliquot of the solution of chelates added to the xylenol solution, which corresponds to only 3% of the total Gd(III) added (9.4 µM). Thus, nearly 97% of gadolinium ions were successfully incorporated by the DOTA-NHS ligand.

### *In vitro* transmetallation assay

To be considered safe for *in vivo* applications, gadolinium-based chelates must be, among other requirements, kinetically inert regarding the transmetallation process between the Gd(III) ion and other endogenous ions, such as Zn(II), Ca(II), iron, and/or copper ions. However, *in vivo*, the presence of Zn(II) ions may displace a more significant amount of Gd(III) ions, since their endogenous concentration is relatively high and they have a high association constant affinity with DOTA-type ligands, when compared with the other metal ions present in the body. The nonchelated Gd(III) retained in body tissues can serve as a source of toxicity and has recently been linked with a medical condition known as nephrogenic systemic fibrosis (NSF), a rare but potentially harmful side effect observed in some patients with severe renal disease or following liver transplant^30^. The insoluble gadolinium hydroxide formed at physiological pH, as well as the Zn(II) coordinated to the ligand, can cause some metabolic deregulation when elimination via the renal excretion pathway occurs^31,32^.

**Figure 1:**
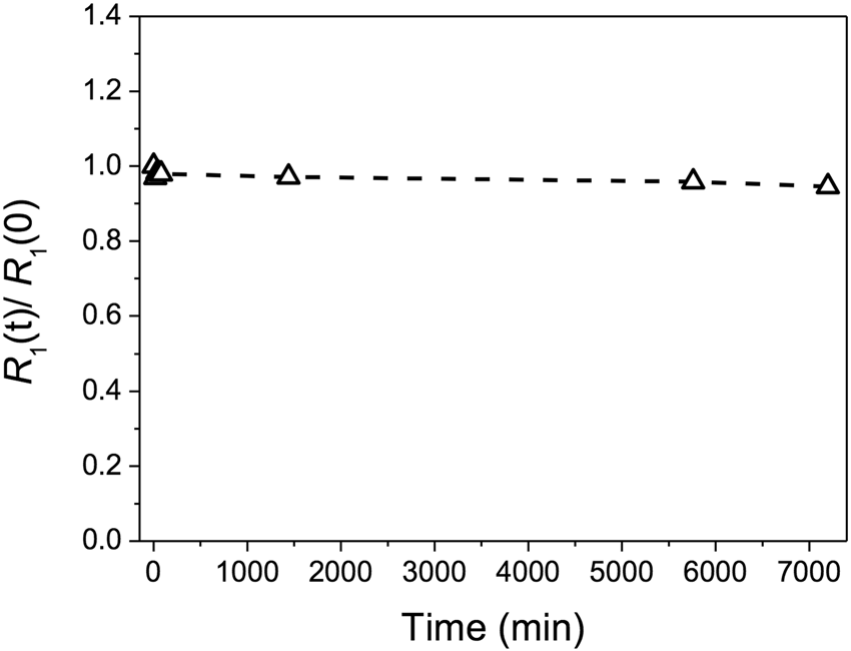
Evolution of *R*_1_(t)/*R*_1_(0) vs. time (min) for the activated Gd(III) chelate measured at 60 MHz and 37 °C.

Our results showed that the activated Gd(III) chelate exhibited negligible change of *R*_1_(t)/*R*_1_(0) values over the 7200 minutes time period monitored meaning non-detectable transmetallation upon exposure to zinc ions in the presence of phosphate ions (Fig. 1). This reflects the high stability of the macrocyclic gadolinium DOTA-based chelate, even after activation by coupling agents, corroborating the work of Laurent *et al*.^31^.

### Optical characterization of QDs and QDs-Gd(III) chelates bimodal nanosystems

**Figure 2:**
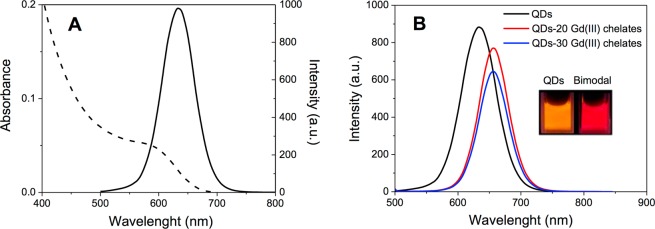
(**A**) Optical characterization of bare CdTe QDs in aqueous suspension: electronic absorption (dashed line) and emission spectra (full line). (**B**) Emission spectra of bare CdTe QDs (black line), QDs-20 Gd(III) chelates and QDs-30 Gd(III) chelates bimodal nanosystems (red and blue lines, respectively). In the picture (inset), we can observe the bright fluorescence of bare QDs (in orange) and of a bimodal nanosystem (in red), under lamp UV excitation at 365 nm. The emission spectra were acquired at λ_exc_ = 488 nm.

QDs suspensions showed a typical electronic absorption profile with a first maximum peak at 591 nm, as observed in Fig. 2A (dashed line). From the spectral analysis we estimated an average diameter of d = 3.4 nm and concentration [QDs] = 2 µM. Furthermore, QDs showed fluorescence in the orange region with maximum at 634 nm and a full width at a half maximum (FWHM) of *ca*. 65 nm (Fig. 2A, full line). The bimodal nanoparticles absorption spectra did not change when compared to the bare QDs (data not shown), whereas the emission spectra, Fig. 2B, showed an average red shift of approximately 22 nm and a slightly narrower FWHM (*ca*. 55 nm) for both QDs-Gd(III) chelates bimodal nanosystems (20 or 30 – prepared as illustrated in Fig. 6). This spectral behaviour is related to the QDs surface alteration during the conjugation process^33^; nevertheless, the bimodal nanoparticles retained a bright fluorescence, as can be observed in Fig. 2B inset. These overall results showed that the conjugation process did not induce considerable changes in the QD emission intensity, making them potential luminescent probes.

### FCS results

**Figure 3:**
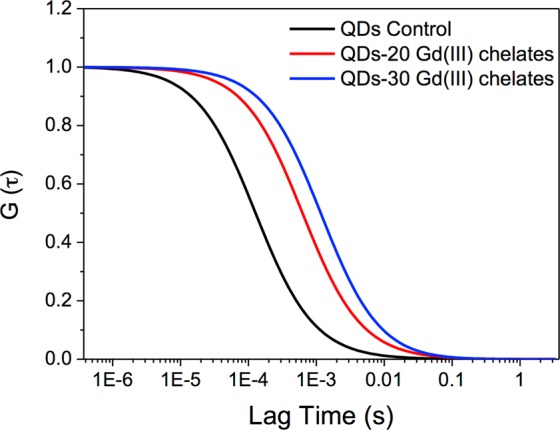
FCS correlation curves of bare QDs (black line), QDs-20 Gd(III) chelates (red line) and QDs-30 Gd(III) chelates (blue line) bimodal nanosystems.

According to the correlation curves shown in Fig. 3, the average diffusion times (τ_D_) were calculated as approximately 127 µs for bare QDs, 646 µs for QDs-20 Gd(III) chelates and 1,141 µs for the QDs-30 Gd(III) chelates bimodal nanosystem. Using these results together with Eq. 1, we obtained the hydrodynamic diameter of the samples *ca*. 3.1 nm for bare QDs and *ca*. 15.9 and 28.2 nm for the bimodal nanosystem prepared with 20 and 30 chelates, respectively. The size of the bare QDs obtained by FCS is in accordance with the diameters obtained from the absorption spectrum. The high sensitivity of the FCS analysis also allowed us to differentiate the nanosystems with 20 or 30 chelates, since the diffusion times and hydrodynamic diameters were proportional to the quantity of ligands expected. Thus, our FCS results also indicate that QDs were successfully conjugated with Gd(III) chelates.

### Relaxometric characterization

The longitudinal (*T*_1_) and transverse (*T*_2_) relaxation time measurements of bulk water protons in the presence of the bimodal nanosystems QDs-Gd(III) chelates are shown in Tables 1 and 2, respectively. From the *T*_1_ and *T*_2_ values, we obtained the relaxivities per Gd(III) and per QD (using Eq. 2), which are also presented in the same Tables. The amount of gadolinium ions was determined by inductively coupled plasma optical emission spectrometry (ICP-OES). According to ICP data (Tables 1 and 2) and the estimated QD concentration, we were able to get an average number, approximately of the order of 15 or 25 chelates per QD, which represents a conjugation yield higher than 75% (the expected QD/chelates ratio was 1/20 or 1/30, respectively, according to the activation procedure applied), showing the efficiency of the methodology for conjugation process performed in this work. A further confirmation of QDs-chelates conjugation obtained by Fourier Transform Infrared spectroscopy can be found in ESI† (Fig. S2).

**Table 1:** Longitudinal relaxation times (*T*_1_) of bulk water protons in the presence of bimodal nanosystems, concentration of Gd(III), and the respective relaxivities given per QDs and per Gd(III).

QDs-Gd(III) chelates	Frequency and Temperature	*T*_1_ (ms)*	Relaxivity per QDs (mM^−1^·s^−1^)	Gd(III) Concentration (x 10^−3^ mM)	Relaxivity per Gd(III) (mM^−1^·s^−1^)
1/20	20 MHz – 25 °C	550	744	35.8	42
	20 MHz – 37 °C	560	753		42
	60 MHz – 37 °C	890	422		24
1/30	20 MHz – 25 °C	440	971	54.6	36
	20 MHz – 37 °C	440	996		36
	60 MHz – 37 °C	720	554		20

^*^An error of *ca*. 10% was evaluated for the *T*_1_ measurements. The concentration of Gd(III) was obtained by ICP, as described in the Experimental Procedure section. The final QDs concentration, obtained from the absorption spectrum, was of approximately 2 μΜ in all the systems. Diamagnetic relaxation rates (*R*_1_) were 0.28 s^−1^ for 20 and 60 MHz (37 °C) and 0.33 s^−1^ for 20 MHz, 25 °C.

**Table 2:** Transverse relaxation times (*T*_2_) of bulk water protons in the presence of bimodal nanosystems, concentrations of Gd(III), and the respective relaxivities given per QDs and per Gd(III).

QDs-Gd(III) chelates	Frequency and Temperature	*T*_2_ (ms)*	Relaxivity per QDs (mM^−1^·s^−1^)	Gd(III) Concentration (x 10^−3^ mM)	Relaxivity per Gd(III) (mM^−1^·s^−1^)
1/20	20 MHz – 25 °C	440	906	35.8	51
	20 MHz – 37 °C	450	851		48
	60 MHz – 37 °C	410	960		54
1/30	20 MHz – 25 °C	360	1,159	54.6	42
	20 MHz – 37 °C	360	1,129		41
	60 MHz – 37 °C	290	1,464		54

^*^An error of *ca*. 10% was evaluated for the measurements of the *T*_2_. The concentration of Gd(III) was obtained by ICP, as described in the Experimental Procedure section. The final QDs concentration, obtained from the absorption spectrum, was of approximately 2 μΜ in all the systems. Diamagnetic relaxation rates (*R*_2_) were 0.52 s^−1^ for 20 and 60 MHz (37 °C) and 0.46 s^−1^ for 20 MHz, 25 °C.

Table 1 shows that the longitudinal relaxivities *r*_1_ per Gd(III) ion for the two bimodal nanosystems were quite similar, being both 5 to 6 times higher than the *r*_1_ of our free activated chelate Gd(III)-DOTA-NHS (*ca*. 4 mM^−1^·s^−1^) at the clinical field of 60 MHz, 37 °C. As expected, the *r*_1_ value for Gd(III)-DOTA-NHS was very similar to Gd(III)-DOTA (Dotarem^**®**^)^28,34,35^. At 20 MHz and 37 °C, our bimodal nanosystems had an *r*_1_ 9 to 10 times higher than Dotarem^®^.

The paramagnetic relaxation process in Gd(III) complexes is depicted according to a model considering (i) the inner sphere (IS) contribution, related to the exchange between the bound water molecules and (ii) bulk water and the outer sphere (OS) contribution, resulting from water molecules diffusing near the paramagnetic metal center. The search for more effective CAs mainly involves the optimization of the main parameters that control the inner sphere *r*_1_ relaxivity: the number of water molecules coordinated to the metal ion (*q*), its exchange lifetime (*τ*_M_) with bulk water and the rotational correlation time (*τ*_R_) of the complex^36^. Thus, one of the approaches used to increase *r*_1_ in the 10–60 MHz frequency range is to reduce the mobility (consequently increasing τ_R_) of paramagnetic chelates, by attaching them to nanoparticles that are, at least, one order of magnitude larger than the chelates. However, the water exchange rate becomes important in limiting the *r*_1_ increase when the rotational motion is slowed^36^.

In the present bimodal nanosystems, although one of the carboxyl arms of the DOTA ligand was used in the chelate conjugation process, leading to an amide group, it is known from many studies with Gd(III)-DO3A monoamide derivatives that the amide oxygen atom still coordinates the Gd(III) ion, keeping the value of *q* = 1^36,37^. In principle, the water exchange rate (*k*_ex_ = 1/*τ*_M_) was not significantly altered in the immobilized chelate relative to the free Gd(III)-DOTA-NHS, due to the maintenance of the unrestricted access of the bulk water molecules to the chelate, which occurs at the external surface of the derivatized QD. Thus, the bimodal nanosystem, having monoamide bonds, is expected to have a relatively low *k*_ex_^298^ value, close to that reported for Gd(III)-DO3A-bz-NO_2_ (1.6 × 10^6^ s^−1^)^38^, which is even lower than the value calculated for Gd(III)-DOTA^34^. This slow water exchange probably limits the *r*_1_ increase expected from the slow rotational motion of the QD-conjugated chelates. We should also consider the relatively high flexibility of the linker (diamine) used for paramagnetic chelates conjugation to the QD surface, which can also limit the relaxivity, because in the rotational motion, both the global tumbling of the immobilized chelate (global *τ*_R_) and its internal motion ability (local *τ*_R_) must be taken into account, according to the Lipari-Szabo treatment^39,40^. Despite the possible negative effects related to the high diamine flexibility and slow *k*_ex_, the *r*_1_ relaxivity values per Gd(III) of our systems were quite high, for which the QD surface, especially for the 1/30 nanosystem, allowed the attachment of an average of 25 Gd(III) chelates per nanoparticle, which results in a higher relaxivity per QD that could represent a lower dosage required for MRI.

At the clinical field strength corresponding to 60 MHz, the dominant correlation time for *r*_1_ is the rotational diffusion, which governs the IS contribution^41^ and, for a slower *τ*_R_, a higher *r*_1_ is observed. However, previous studies with different gadolinium chelates showed that if *τ*_R_ is slowed significantly (for instance when molecular chelates are bound to proteins), *r*_1_ decreases at higher magnetic fields with increasing *τ*_R_. Thus, *τ*_R_ of our nanoparticulate bimodal systems is slow enough to decrease *r*_1_ at 60 MHz when compared to 20 MHz.

Values of transverse relaxivities *r*_2_, at different temperatures and magnetic field strengths, are also shown in Table 2. From this data, we can observe that the *r*_2_/*r*_1_ ratios for both systems increased from 1.1 at 20 MHz up to 2.7 at the clinical field of 60 MHz, due to the expected decrease of *r*_1_ and increase of *r*_2_ at 60 MHz when compared to 20 MHz^38^. Thus, our bimodal nanosystems are promising *T*_1_-weighting CAs, since the *r*_2_/*r*_1_ ratios are small enough. The linear behavior of longitudinal relaxivity rates (*R*_1_) *versus* gadolinium concentrations is also exemplified in Fig. S3 (ESI**†**) for the bimodal nanosystem 1/30 (at 60 MHz, 37 °C). Additionally, *T*_1_-weighted MR images related to our bimodal nanosystem, which were acquired at 9.4 T, are depicted in Fig. S4 (ESI†).

Bimodal systems based on QDs conjugated to Gd(III) chelates have been previously reported, looking not only for a dual signal but also for better relaxivities in comparison to the molecular CAs used clinically^24–26,42,43^. Stasiuk *et al*.^24,42^ have prepared a series of interesting systems through covalent attachment of Gd(III) complexes of a pyridine-based chelating ligand (DPAA), with a *q* value of 3, onto hydrophobic InP/ZnS QDs using linkers of different flexibilities, and thus obtaining a *r*_1_ up to 31.5 mM^−1^·s^−1^ per Gd(III) (at 35 MHz and 25 °C)^24^. In our case, we obtained *r*_1_ values up to 42 mM^−1^·s^−1^ per gadolinium ion (at 20 MHz and 37 °C), which are equivalently efficient, using more stable cyclic Gd(III)-DOTA-type chelates and hydrophilic QDs, prepared by a simple route that did not require post-synthesis procedures to reach the hydrophilicity necessary to work with biological systems.

Jin *et al*.^43^ have also prepared a system with a Gd(III)-DOTA-type chelate bound to the surface of glutathione stabilized CdSeTe/CdS QDs, previously prepared in organic medium followed by ligand exchange. This system showed a value of 365 mM^−1^.s^−1^ per nanoparticle (500 MHz, 25 °C), which could be higher at lower fields. However, *r*_1_ per ion was not reported. Recently, McAdams and colleagues (2017) obtained a system with the highest relaxivity of 6800 mM^−1^·s^−1^ per nanoparticle (42.5 MHz, 27 °C) due to the bidentate attachment of *ca*. 620 magnetic centers, Gd(III) complexes of a DTPA-bisamide of *p*-aminothiophenol, per multishell CdSe/CdS/ZnS QD, although with a relatively low *r*_1_ relaxivity per Gd(III) (*r*_1_ = 11 mM^−1^·s^−1^)^25^.  Furthermore, the nanoprobe presented drastic quenching of the emission intensity, different from our bimodal nanosystems that remained highly fluorescent. In 2017, Yang and co-workers also described the synthesis and biological application of a bimodal CA developed by chelation of the Gd(III) ion to the linear DTDTPA-modified CuInS_2_/ZnS QDs. Besides the hydrophobicity of the applied QDs, the *r*_1_ of the resulting nanoprobe was just 2.5 times higher that of clinically approved linear Gd(III)-DTPA^26^.

Thus, compared to previous studies, our bimodal nanosystems combine interesting features, such as direct conjugation approach with stable Gd(III)-DOTA derivative chelates on as-prepared hydrophilic CdTe QD surfaces, very intense fluorescence, and effective longitudinal relaxivities per QD and per paramagnetic ion, at least 5 times [per Gd(III)] and 100 times (per QD) higher than the *r*_1_ for Gd(III)-DOTA chelates. Thus, our bimodal nanosystems show a high potential to be useful in fluorescent assays and as positive contrast agents in *T*_1_-weighted MRI. Additionally, we also showed that our systems are stable in the presence of other metal ions by the transmetallation assay, a characterization that is missing in most of the studies of this kind found in the literature.

### Biological interactions of the bimodal systems

#### Resazurin viability assay

**Figure 4:**
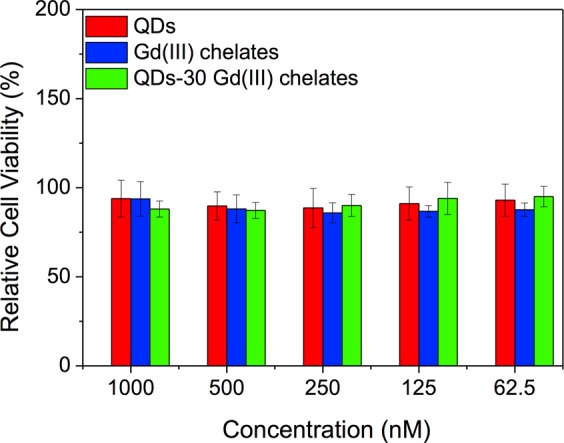
Relative cell viability analysis using the resazurin assay. HeLa cells remained viable after being incubated with 62.5 to 1000 nM of bare QDs, bare Gd(III) chelates and QDs-30 Gd(III) chelates. Results were statistically non-significant with p > 0.05.

The resazurin assay offered a simple and sensitive cytotoxicity evaluation of the bimodal nanosystems based on QDs and Gd(III) chelates, applied to HeLa cells. We used HeLa cells as a proof of concept, since they are frequently used as a model in studies related to cancer, which also is one of the main targets motivating the development of bimodal nanosystems^33^. This assay indicates whether the cells are metabolically active, and in this case, being able to reduce resazurin (oxidized form) to resorufin (reduced form). We found that the bimodal systems developed by us presented low toxicity, as can be observed by the relative percentage of cell viability, calculated by Eq. 3, in Fig. 4: 88.0 ± 4.5% [1000 nM of QDs-30 Gd(III) chelates] to 95.1 ± 5.8% (62.5 nM). The maximum concentration tested in the viability assay was ten times higher (1000 nM) than the one applied in the labeling procedure (100 nM), and it was not sufficient to induce cytotoxic effects on cells under these conditions. This assay was performed using the bimodal nanosystem with the higher chelate amount. Also, neither bare QDs nor paramagnetic chelates showed a considerable cytotoxicity to HeLa cells, with cell viability higher than *ca*. 85%. Results of this assay were statistically non-significant with p > 0.05. The positive control, using dimethyl sulfoxide (DMSO), induced a 100% of HeLa cell death (data not shown).

Su *et al*.^44^ also studied the toxicity of CdTe QDs by the MTT assay in concentrations up to 300 nM, which showed to be toxic to human embryonic kidney cells (HEK293). Even testing higher concentrations than the authors above, our results showed that the cells remained viable. We believe that the purification procedure applying an ultrafiltration column was an important step to reach this result, since it decreases the presence of the reagents used in excess in the synthesis and conjugation procedures. In the viability assays that directly evaluate the cells, such as MTT, the optical properties of the internalized QDs can interfere with the viability data, leading to false positive results, principally at high QD concentration^45^. This is one of the reasons why we choose the resazurin assay as an alternative, in order to eliminate the interferences on the QD optical properties, since it uses the supernatant without QDs in the analysis.

#### Confocal fluorescence microscopy

**Figure 5:**
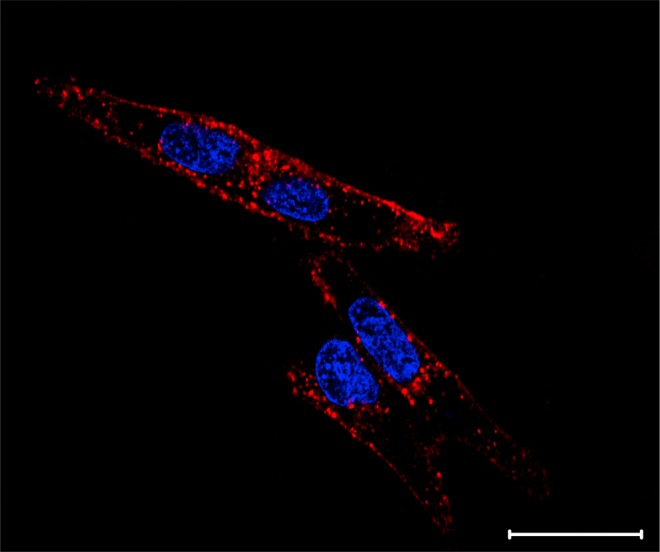
Confocal fluorescence microscopy images of HeLa cells non-specifically labeled by the bimodal nanosystem (in red). The cell nuclei are stained in blue by Hoechst. Scale Bar: 20 µm.

According to the confocal fluorescence microscopy images, bimodal nanosystems labeled efficiently HeLa cells by unspecific interaction, as can be seen in Fig. 5.

Probably, this labeling was due to the remaining QDs carboxylic groups activated by NHS esters, which were not conjugated to Gd(III)-DOTA-NHS, that allow the interaction of these nanoparticles with the amine groups of the membrane proteins and/or to the endocytosis of the QDs by the cells. We observed that both prepared nanosystems presented a similar labeling profile. Therefore, these systems were able to label the cells efficiently, presenting a bright optical signal, indicating that our nanosystems can be used as useful probes for optical imaging.

## Experimental Section

### Gd(III) chelates preparation

Gd(III) chelates were prepared using commercial DOTA (1,4,7,10-tetraazaciclododecano-1,4,7,10-tetraacetic acid – Sigma-Aldrich) as ligand and a gadolinium chloride solution (GdCl_3_.6H_2_O–Alfa Aesar), with a 10% stoichiometric excess of ligand. Firstly, the pH of a DOTA ligand solution (10 µmol) was adjusted to 5.5 with NaOH (1 M), which was followed by the activation of one carboxyl group of each DOTA, by using *N*-ethyl-*N*′-(3-dimethylaminopropyl) carbodiimide hydrochloride (EDC – Sigma-Aldrich) and of *N*-hydroxysulfosuccinimide sodium salt (Sulfo-NHS – Sigma-Aldrich)^46–49^, both at 10 µmol. After this procedure, gadolinium ions (9 µmol) were added to the solution of the previously activated ligand (DOTA-NHS), which was kept under overnight stirring at approximately 25 °C to obtain an amine-reactive complex Gd(III)-DOTA-NHS with a final concentration of 10 mM. The efficiency of the coordination and the stability of the complex were evaluated, respectively, by the xylenol orange and the transmetallation assays.

### Coordination efficiency evaluation by the xylenol orange assay

The efficiency of the paramagnetic chelate formation was evaluated using the xylenol orange assay proposed by Barge and colleagues^29^. By correlating changes of xylenol orange absorbances with different Gd(III) concentrations in a UV-Vis 1800 spectrophotometer (Shimadzu), we obtained a calibration curve (Fig. S1 – ESI**†**), which was used to evaluate the amount of free gadolinium ions in the paramagnetic chelate solution. For this, an amount of 4 µL of a chelate solution was added to 2 mL of the xylenol solution to reach a final concentration of 9.4 µM Gd(III).

### *In vitro* transmetallation assay

When Gd(III) chelates are applied in biological systems, exchange of gadolinium ions with endogenous metal ions, such as Zn(II), can occur through transmetallation. This process is used to evaluate the kinetic stability of the prepared chelate, which is performed by analyzing the evolution in time of the paramagnetic longitudinal relaxation rate (*R*_1_) of water protons of a Gd(III) chelate solution upon addition of Zn(II) at physiological conditions (pH = 7.0 and 37 °C). The paramagnetic relaxation rate (*R*_1_) is obtained after subtracting the diamagnetic contribution of the proton water relaxation (${R}_{1}^{dia}=1/{T}_{1}^{dia}$) from the observed relaxation rate (${R}_{1}^{obs}=1/{T}_{1}^{obs}$). Thus, an aliquot of the Gd(III) chelate solution was diluted to 2.5 mM in phosphate buffer ([KH_2_PO_4_] = 0.026 M, [Na_2_HPO_4_] = 0.041 M), in the presence of a 2.5 mM zinc chloride solution. Then, *R*_1_ values were obtained by measuring ${T}_{1}^{obs}$ over time (0 to 7200 min) in a Bruker Minispec mq60 relaxometer (60 MHz, 1.5 T, 37 °C), according to the procedure reported by Laurent *et al*.^31^.

### Synthesis of CdTe-MSA QDs

Cadmium Telluride (CdTe) QDs were synthesized in an aqueous medium, using 3-mercaptossuccinic acid (MSA) as functionalizing/stabilizing agent in a molar ratio of 5:1:6 (Cd:Te:MSA), according to a previously reported method^33^. Briefly, metallic tellurium (0.1 mmol – Sigma-Aldrich) was reduced by reaction with sodium borohydride (NaBH_4_ – 3 mmol – Sigma-Aldrich) at high pH (>10) and inert atmosphere (N_2_). Te^2-^ was added to a previously prepared solution of Cd^2+^/MSA [0.5 mmol of cadmium chloride (CdCl_2_ – Alfa Aesar) and 0.6 mmol of MSA (Sigma-Aldrich)] at pH ~ 10.5. QDs suspension remained for 7 h under constant stirring at 90 °C to obtain a red emission.

### Bimodal nanosystems preparation

**Figure 6:**
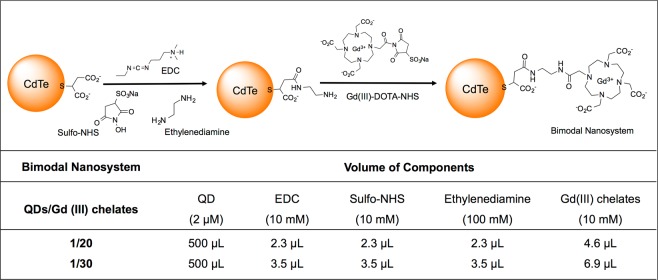
Top: Schematic representation of the conjugation process between QDs and Gd(III)-DOTA-NHS chelates using EDC, Sulfo-NHS, and ethylenediamine. Bottom: description of the component volumes added to each bimodal nanosystem.

Bimodal nanosystems based on QDs and Gd(III) chelates were obtained using a covalent coupling procedure^46^. To achieve an improved bimodal system, various molar ratios of QDs/Gd-DOTA-NHS were tested, from 10 to 100 Gd(III) chelates per QD. The systems that remained visually stable, with bright fluorescence, without precipitation, were the ones prepared to reach 20 or 30 Gd(III) chelates per QD. Therefore, only these systems were characterized and further studied. The systems were prepared by mixing the following chemical compounds: EDC (10 mM), Sulfo-NHS (10 mM), ethylenediamine (100 mM) and Gd(III)-DOTA-NHS solution (10 mM), as schematized in Fig. 6. Firstly, the pH of 0.5 mL of carboxyl-coated CdTe QDs aqueous suspensions (2 µM) was adjusted to 5.5 using a MSA solution (4.9% w/v). Then, the QD suspension was ultra-filtrated using a 10 kDa Vivaspin column to remove excess of free stabilizing agent. After this, the respective aliquots of coupling agents (EDC and Sulfo-NHS) were added to obtain a QD-NHS ester suspension. The QDs-NHS were reacted with ethylenediamine by overnight shaking and ultra-filtered to remove excess of ethylenediamine. Afterwards, Gd(III)-DOTA-NHS chelates were added in twice the amount established for each nanosystem, to guarantee a high conjugation yield. Before characterization and application, the nanosystems were also ultra-filtered to remove any eventual remaining reactants.

### QDs and bimodal systems optical characterization

Bare QDs and the bimodal nanosystems were characterized by electronic absorption and emission spectroscopy, using respectively, a UV-Vis 1800 spectrophotometer (Shimadzu) and a LS55 spectrofluorometer (PerkinElmer, λ_exc_ = 488 nm). The QD mean diameter was estimated considering the wavelength at the first maximum absorption peak and the empirical equation proposed by Dagtepe *et al*.^50^. Then, the QD suspension concentration was estimated using the Lambert–Beer’s law, considering the molar particle extinction coefficient (ε) of CdTe QDs calculated from the QD mean diameter, according to Yu *et al*.^51^, the absorbance value at the first maximum absorption peak, and the length of the light path in cuvette (1 cm).

### Fluorescence correlation spectroscopy

The FCS analysis was performed in a confocal microscope (LSM 780, Carl Zeiss Meditec AG, Jena, Germany) using 40× water immersion objective (NA = 1.0, WD = 2.5 mm) and a pinhole aperture of 35 µm^52^. Correlation curves were obtained, at 514 nm excitation, for both bare QDs and bimodal systems. From the correlation curves, diffusion times (τ_D_) were obtained for each sample and then the hydrodynamic radius (R) for each sample was calculated by applying Eq. 1:

1 $${\rm{R}}=\frac{4\,{{\rm{k}}}_{{\rm{B}}}{\rm{T}}\,{{\rm{\tau }}}_{{\rm{D}}}}{6{{\rm{\pi }}{\rm{\eta }}{\rm{\omega }}}_{{\rm{x}}}^{2}}$$

where k_B_ is the Boltzmann constant, T is the temperature of the sample under laser irradiation (T ≈ 303 K), τ_D_ is the diffusion time, η is the medium viscosity (from water η = 7.98 × 10^−4^ Pa·s) and ω_x_ is the lateral radius of the focal volume (300 nm for excitation used here – 514 nm). FCS allows the evaluation of the conjugation through the determination of *R* (from τ_D_), since the τ_D_ value is higher for a conjugated sample when compared to bare QD^52^.

### Relaxometric measurements

The relaxometric properties of QDs-Gd(III) chelates bimodal nanosystems were evaluated by measuring the bulk water proton longitudinal (*T*_1_) and/or transverse (*T*_2_) relaxation times on the Bruker Minispec mq60 (1.5 T, 37 °C) and mq20 (0.49 T, at 25 and 37 °C) relaxometers, using inversion recovery and Carr-Purcell-Meiboom-Gill (CPMG) pulse sequences for *T*_1_ and *T*_2_, respectively. The longitudinal and transversal relaxivities (*r*_1,2_) of the bimodal systems were obtained using *T*_1,2_ values and the Gd(III) concentrations (mM), as described by Eq. 2:

2 $${r}_{1,2}=\frac{{R}_{1,2}^{obs}-{R}_{1,2}^{dia}}{{\rm{C}}}$$

where C is the concentration of the paramagnetic ion, ${R}_{1,2}^{obs}$ and ${R}_{1,2}^{dia}$ are the longitudinal or transverse relaxation rate (1/*T*_*1*,*2*_) of the water protons in the presence and absence of the paramagnetic ions, respectively. The relaxivity indicates the efficiency of the CA to decrease the relaxation times of water protons per unit of concentration (mM). In this study, we calculated the relaxivity per gadolinium ion and also per QD. The Gd(III) concentration in the systems were determined using ICP-OES (Thermo Scientific – iCAP 6300). The Gd(III)-DOTA-NHS chelate was also characterized by the same procedure. The linear dependence between the longitudinal bulk water proton relaxation rate (1/*T*_1_), in the presence of bimodal nanosystems and the gadolinium concentration was also evaluated (1.5 T, 37 °C).

### Cell culture

HeLa cells (human epithelial cervical carcinoma) were obtained from ATCC (American Type Culture Collection, Manassas, VA, USA) and cultured in Dulbecco’s modified Eagle’s medium (DMEM – Sigma-Aldrich) supplemented with 10% of fetal bovine serum (FBS - Gibco), streptomycin and penicillin (Sigma-Aldrich) in humidified atmosphere with 5% CO_2_ at 37 °C. When reaching 90% confluence, cells were detached with 0.25% of trypsin (Sigma-Aldrich) and followed by neutralization with DMEM.

### Fluorescence microscopy analysis

The labeling of HeLa cells by the bimodal nanosystems was evaluated using fluorescence microscopy. For this, 3 × 10^4^ cells/well were seeded in a 4-wells plate (Greiner Bio-One, Germany) and incubated for 24 h. After this time, cells were washed and incubated in the following conditions: (I) control cells and (II) cells with QDs-Gd(III) chelates bimodal nanosystems at 100 nM for 1 h (at 37 °C and 5% CO_2_). After the incubation, cells were washed three times with PBS.

Cells were analyzed in a confocal fluorescence microscope (Zeiss LSM780-NLO) under 488 nm excitation to evaluate the labeling of HeLa cells by the bimodal QDs-Gd(III) chelate systems. Moreover, the excitation at 800 nm (NLO Ti: Saphira Mai Tai) was applied to display the cell nuclei stained by Hoechst. For this, cells were incubated for 5 min at a final concentration of 1 μg.mL^−1^ ^33^.

### Resazurin viability assay

Cell viability was evaluated for QDs, Gd(III) chelates and bimodal QDs-Gd(III) chelates at concentrations of 1000, 500, 250, 125, and 62.5 nM in triplicate in two independent assays. PBS (50% v/v in DMEM) was used as negative control, since all nanosystems were diluted in PBS. DMSO (20% v/v in DMEM) was used as positive control. For this assay, the QD suspension was also ultra-filtrated using a 10 kDa Vivaspin column. For this, 5 × 10^4^ cells/well were seeded in a 24-wells plate (Greiner Bio-One, Brazil) and incubated for 24 h. Afterwards, wells were washed and cells were incubated for more 24 h with the samples, at 37 °C. The cell activity was revealed by the resazurin reduction obtained after approximately 40 min of incubation^53^. Thus, the supernatant was removed, wells were washed and the amount of 10 mg·mL^−1^ of the resazurin was added to the wells. Finally, after 40 min of incubation, the supernatant was added in a 96-wells plate to measure the absorbance of resorufin at 570 nm and of resazurin at 600 nm in a μQuantTM microplate spectrophotometer (BioTek, Inc.) equipped with Gen5 2.06 software. Results were evaluated by one-way ANOVA, followed by the Tukey post hoc test for the different concentrations. The cell viability was calculated using Eq. 3:

3 $${\rm{cell}}\,{\rm{viability}}\,( \% )=\frac{({{\rm{A}}}_{570}-{{\rm{A}}}_{600})\,{\rm{treated}}\,{\rm{cells}}}{({{\rm{A}}}_{570}-{{\rm{A}}}_{600})\,{\rm{control}}\,{\rm{cells}}}\times 100$$

## Conclusions

We developed new fluorescent/paramagnetic bimodal nanosystems consisting of hydrophilic CdTe QDs efficiently functionalized with stable Gd(III)-DO3A monoamide chelates by direct conjugation, without requiring laborious experimental procedures. Our nanosystems showed effective longitudinal relaxivities per QD and per paramagnetic ion, at least 5 times [per Gd(III)] and 100 times (per QD) higher than the *r*_1_ for Gd(III)-DOTA chelates, suitable for inducing a positive contrast in *T*_1_-weighted MR images. Importantly, our bifunctional nanoparticles also preserved an intense fluorescence, successfully labeling HeLa cells, with negligible cytotoxicity, under the conditions applied, in a 24 h assay. It is also worth to mention that the nanosystems showed great colloidal stability, intense fluorescence and kept the relaxivity value for at least three months. Thus, our systems can be considered as attractive bimodal nanoprobes, versatile to be conjugated to biomolecules with different specificities, opening new possibilities for performing a variety of biological studies, at molecular and cellular levels, by applying fluorescence and magnetic resonance assays.

## Supplementary information

Electronic Supporting Information (ESI†)

## Data Availability

All data generated or analysed during this study are included in this published article (and in its Supplementary Information file).
