# Supplementary material for: Hydrophilic Quantum Dots Functionalized with Gd(III)-DO3A Monoamide Chelates as Bright and Effective T1-weighted Bimodal Nanoprobes
Source: Sci Rep. 2019 Feb 20;9:2341. doi: 10.1038/s41598-019-38772-8 (PMC6382838; doi:10.1038/s41598-019-38772-8)
Supplement: Supplementary file 1 — Electronic Supporting Information (ESI†) [file 41598_2019_38772_MOESM1_ESM.pdf]

Electronic Supporting Information (ESI†)

Hydrophilic Quantum Dots Functionalized with Gd(III)-DO3A Monoamide Chelates as Bright and Effective T_1-weighted Bimodal Nanoprobes

Maria I. A. Pereira[1], Goreti Pereira[2], Camila A. P. Monteiro[1], Carlos F. G. C. Geraldes[3,4], Paulo E. Cabral Filho[1], Carlos L. Cesar[5,6], André A. de Thomaz[6], Beate S. Santos[7], Giovannia A. L. Pereira[2,*,#], Adriana Fontes[1,*,#]

[1] Departamento de Biofísica e Radiobiologia, Universidade Federal de Pernambuco, Recife, PE, Brazil.

[2] Departamento de Química Fundamental, Universidade Federal de Pernambuco, Recife, PE, Brazil.

[3] Departamento de Ciências da Vida, Faculdade de Ciência e Tecnologia, Universidade de Coimbra, Coimbra, Portugal.

[4] Centro de Química de Coimbra, Universidade de Coimbra, Coimbra, Portugal.

[5] Departamento de Física, Universidade Federal do Ceará, Fortaleza, CE, Brazil.

[6] Departamento de Eletrônica Quântica, Instituto de Física Gleb Wataghin, Universidade Estadual de Campinas, Campinas, SP, Brazil.

[7] Departamento de Ciências Farmacêuticas, Universidade Federal de Pernambuco, Recife, PE, Brazil.

*Shared senior authorship.

[#]**Corresponding authors:** Giovannia A. L. Pereira, Av. Jornalista Aníbal Fernandes, S/N, Departamento de Química Fundamental, CCEN, UFPE, 50740-560, Recife, PE, Brazil - Phone: +55 81 21268444, e-mail: giovannia_pereira@yahoo.com; Adriana Fontes, Av. Prof. Moraes Rego, S/N, Departamento de Biofísica e Radiobiologia, CB, UFPE, 50670-901, Recife, PE, Brazil - Phone: +55 81 21267818, e-mail: adriana.fontes.biofisica@gmail.com.

1. Xylenol Orange Assay

The xylenol orange solution is yellow either in the acid or neutral pH, and when the pH becomes basic, its color changes to violet. This effect is due to deprotonation of the phenolic hydroxyl group, which also occurs in the presence of coordination metals, such as the Gd(III). As consequence, a shift of the absorption wavelength to higher values is observed in the presence of free Gd(III) and the solution becomes violet.[1]

Briefly, it was prepared an acetate buffer solution (250 mL), the pH was adjusted to 5.8 with NaOH (2 M) and the xylenol orange (3 mg) was dissolved. The spectrophotometric changes of xylenol orange absorptions observed in Figure S1A were recorded by using xylenol orange and $GdCl_3$ solutions (0.001 M) in the following Gd(III) concentrations: 0, 0.1, 0.5, 1, 1.5 and 2 μM. By relating the maxima of these absorbance profiles in the presence of different concentrations of Gd(III), we obtained the calibration curve (Figure S1B). Free Gd ions in solution were determined using this calibration curve. The acetate buffer solution was used as the reference in the analysis.

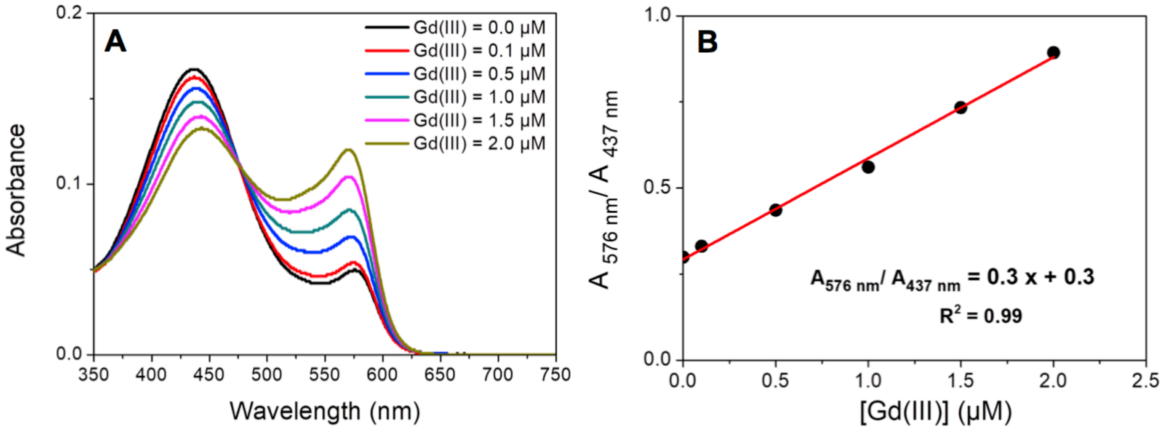

Figure S1. A) Determination of free Gd(III) by xylenol orange. With the increase of the amount of free gadolinium, there is a decrease in the intensity of the peak 437 nm and an increase of the 576 nm. Spectra were recorded by using an acetic buffer solution at pH 5.8 in the presence of 0, 0.5, 1, 1.5 and 2 μM of Gd(III). B) A calibration curve obtained by spectrophotometric changes of xylenol orange absorptions in the presence of different concentration of Gd(III).

2. Fourier Transform Infrared (FTIR) analyses

Vibrational spectra for QDs and the bimodal nanosystem (1/30) were acquired by a Fourier Transform Infrared spectroscopy coupled with attenuated total reflectance (FTIR-ATR) on a Bruker IFS66 spectrophotometer. For this, samples were concentrated in a SpeedVac (RVC 2-18 CDplus - Christ) for about 2 hours.

By analyzing the vibrational modes presented in the spectra depicted in Figure S2, we found evidence of the chelate binding to the QDs. The vibrational spectrum of QDs has been already described in the literature[2] and show the characteristic vibrational modes of the alkyl thiol molecules, that is, (i) νCH_2 at 2955-2880 cm^{-1}, (ii) two νCOO^- at 1578 and 1390 cm^{-1}, and (iii) νC-O at 1072 cm^{-1}. After the chelate conjugation, we observed the appearance of the νC=O mode at 1749 cm^{-1} and a substantial increase of the C-O vibration, which can be interpreted as the overlap of the C-N (at 1065 cm^{-1}) considering that we added 6 C-N bonds to the ensemble. Moreover, the CH_2 vibrational modes bound to the nitrogen atom are slightly shifted to lower energy (2924 – 2865 cm^{-1}) reflecting the 12 N-CH_2 vibrational modes.

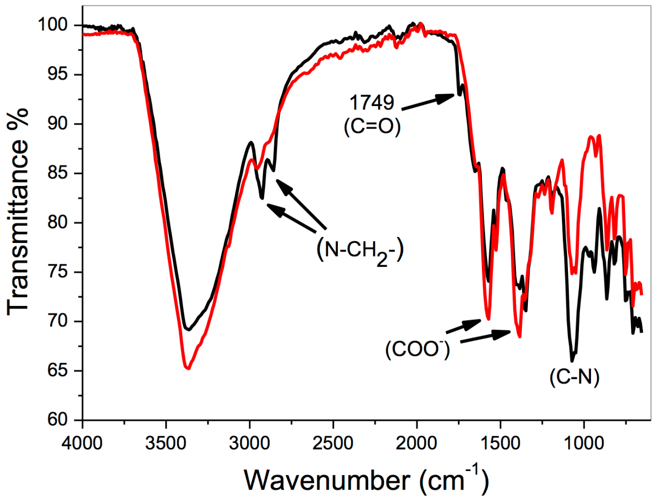

Figure S2. Fourier Transform Infrared spectra for QDs (in red) and the bimodal nanosystem (in black).

3. Longitudinal Relaxometric Characterization of Bimodal Nanosystems

Figure S3 shows the linear dependence between the inverse proton longitudinal relaxation time (R_1) for the 1/30 bimodal nanosystem. For this, T_1 values were measured in a Bruker Minispec mq60 relaxometer (60 MHz, 1.5 T, 37 °C), as a function of different paramagnetic bimodal nanosystem concentrations. A relaxivity of *ca.* 20 $mM^{-1}.s^{-1}$ for this system was obtained.

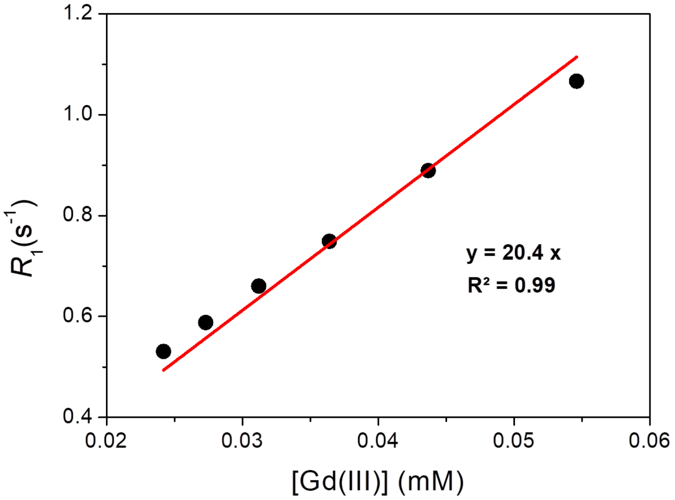

Figure S3. Relaxation rate over different concentrations of Gd(III) from the 1/30 bimodal nanosystem at 1.5 T and 37 ˚C.

4. T_1-weighted Magnetic Resonance Imaging

Magnetic resonance imaging (MRI) of the bimodal nanosystem (1/30) and the control (phosphate buffered saline 1X – PBS), inserted in glass tubes, were carried out, at 25 ^{o}C, in a Varian VNMRS 400 MHz (9.4 T) nuclear magnetic resonance (NMR) spectrometer operating at 399.8 MHz (^{1}H) by using a micro-image probe. The sequence of images was obtained using a SEMS protocol (Spin Echo Multi Slice). T_1 measurements, from imaging, were performed using an inverse recovery pulse sequence with repetition and echo times proper for the signal acquisition. T_1 data were analyzed by MATLAB.

Figure S4 shows the T_1-weighted MR images of PBS (T_1 ~ 2 s) and the bimodal system (T_1 ~ 0.9 s; r_1 ~ 11 $mM^{-1}.s^{-1}$). Even at this high magnetic field, it was possible to observe a

contrast, where the black (or dark blue) image corresponds to PBS, while the bimodal system is the brighter (or light green) one. This behavior indicates that the bimodal nanosystem is an effective nanoprobe that can be used in clinical fields, a magnetic region in which the contrast difference is higher due to the mechanisms related to the paramagnetic relaxation that contribute to the increment of r_1, as previously reported by the literature.[3]

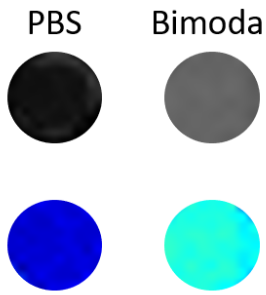

Figure S4. T_1-weighted MR images (at 9.4 T) of PBS (in black and dark blue) and the bimodal nanosystem (in gray and light green).

References

1. Barge, A., Cravotto, G., Gianolio, E. & Fedeli, F. How to determine free Gd and free ligand in solution of Gd chelates. A technical note. *Contrast Media Mol. Imaging* **1,** 184–188 (2006).

2. Ayyaswamy, A., Ganapathy, S., Alsalme, A., Alghamdi, A. & Ramasamy, J. Structural, optical and photovoltaic properties of co-doped CdTe QDs for quantum dots sensitized solar cells. *Superlattices Microstruct.* **88**, 634–644 (2015).

3. Tóth, É., Helm, L. & Merbach, A. Relaxivity of Gadolinium(III) Complexes: Theory and Mechanism. In *The Chemistry of Contrast Agents in Medical Magnetic Resonance Imaging* 25–81 (John Wiley & Sons, Ltd, 2013).
